# LINE-1 and Alu hypomethylation in mucoepidermoid carcinoma

**DOI:** 10.1186/1472-6890-13-10

**Published:** 2013-03-19

**Authors:** Porntipa Sirivanichsuntorn, Somboon Keelawat, Kittipong Danuthai, Apiwat Mutirangura, Keskanya Subbalekha, Nakarin Kitkumthorn

**Affiliations:** 1 Department of Oral and Maxillofacial Surgery, Faculty of Dentistry, Chulalongkorn University, Bangkok 10330, Thailand; 2 Department of Pathology, Faculty of Medicine, Chulalongkorn University, Bangkok 10330, Thailand; 3 Department of Oral Pathology, Faculty of Dentistry, Chulalongkorn University, Bangkok 10330, Thailand; 4 Center of Excellence in Molecular Genetics of Cancer and Human Diseases, Department of Anatomy, Faculty of Medicine, Chulalongkorn University, Bangkok 10330, Thailand; 5 Department of Oral and Maxillofacial Pathology, Faculty of Dentistry, Mahidol University, Bangkok 10400, Thailand

**Keywords:** Mucoepidermoid carcinoma (MEC), Methylation, Long INterspersed Element-1s (LINE-1s), Alu element, Laser capture microdissection

## Abstract

**Background:**

Mucoepidermoid carcinoma (MEC) can be classified into low-, intermediate-, and high-grade tumors based on its histological features. MEC is mainly composed of three cell types (squamous or epidermoid, mucous and intermediate cells), which correlates with the histological grade and reflects its clinical behavior. Most cancers exhibit reduced methylation of repetitive sequences such as Long INterspersed Element-1 (LINE-1) and Alu elements. However, to date very little information is available on the LINE-1 and Alu methylation status in MEC. The aim of this study was to investigate LINE-1 and Alu element methylation in MEC and compare if key differences in the methylation status exist between the three different cell types, and adjacent normal salivary gland cells, to see if this may reflect the histological grade.

**Methods:**

LINE-1 and Alu element methylation of 24 MEC, and 14 normal salivary gland tissues were compared using Combine Bisulfite Restriction Analysis (COBRA). Furthermore, the three different cell types from MEC samples were isolated for enrichment by laser capture microdissection (LCM), essentially to see if COBRA was likely to increase the predictive value of LINE-1 and Alu element methylation.

**Results:**

LINE-1 and Alu element methylation levels were significantly different (*p*<0.001) between the cell types, and showed a stepwise decrease from the adjacent normal salivary gland to the intermediate, mucous and squamous cells. The reduced methylation levels of LINE-1 were correlated with a poorer histological grade. In addition, MEC tissue showed a significantly lower level of LINE-1 and Alu element methylation overall compared to normal salivary gland tissue (*p*<0.001).

**Conclusions:**

Our findings suggest that LINE-1 methylation differed among histological grade mucoepidermoid carcinoma. Hence, this epigenetic event may hold value for MEC diagnosis and prognostic prediction.

## Background

Mucoepidermoid carcinoma (MEC) is a malignant neoplasm of salivary glands that occurs in both adults and children [1-4]. MEC typically occurs in 40–60-year-old patients, and with a median age of approximately 45 years. There is a 3:2 male:female gender preference for MECs with the exception of the tongue and retromolar area, which are more common in females [1,5]. Fifty-three percent of MECs are found in the major salivary glands, especially the parotid glands, while the palate and the buccal mucosa are the most common intraoral sites [1].

Histologically, MECs are primarily composed of three morphological cell types, which include squamous or epidermoid, mucous and intermediate cells, and these can take the form of a solid nest or cystic structure. According to the WHO classification system, MECs are classified as low-, intermediate- or high-grade based on five histological features: the presence of a cystic component, neural invasion, necrosis, mitotic activity and anaplasia [1]. However, many systems have been proposed for grading this tumor type, but none have been universally accepted [1,6-10]. Sadly, the outcome of MEC patients is influenced by the clinical stage and histological grade [11], whereby patients with high-grade, the rate of recurrence and metastasis is increased and thus compromising survival [1,12,13]. Furthermore, it has been suggested that the histological grade of MEC can considerably impact the treatment outcome of affected patients.

To date, only a few genetic studies have proposed mechanisms for the etiology of MEC. Some MECs have been reported to have a t(11:19)(q21:p13) translocation, and abnormality [14-16] that is also shared by acute leukemias [1,17-19]. Furthermore, a study reported that 18% of MECs analyzed demonstrated mutations in *H-ras* gene at codon 12 and/or 13 (and none at codon 61), but however these were essentially detected in high-grade cases [1,20].

One of the most common epigenetic changes found in cancer is the genome-wide decrease in methylation (genome-wide hypomethylation) [21-23]. Long INterspersed Element-1s (LINE-1s) are retrotransposons with highly repetitive, interspersed sequences which are distributed randomly throughout the genome, and constituting 17% of the total human genome [24,25]. Furthermore, Alu represents the most abundant Short INterspersed Element (SINE) repetitive sequence, representing 11% of total human genome [26]. Hypomethylation of LINE-1s, which occurs in many malignancies [21,27-31], generally results in chromosomal aberrations [32-35], hypermethylation, mutations of key tumor suppressor genes [36,37], and changes in oncogene transcription [38] resulting in the altered expression of cancer-related genes [39]. In addition, LINE-1 hypomethylation levels may hold value as a prognostic marker for epithelial solid cancers, for example cervical [30], hepatocellular [31] and ovarian [29]. Similarly, Alu hypomethylation have also been reported for many types of cancers, such as colorectal [27], gastric [28], and hepatocellular [40]. Thus, both LINE-1 and Alu element hypomethylation may play a notable role in different histological feature of cancer.

Most methylation studies report only quantitative information about the methylation level. Recently, we reported that the methylation patterns of LINE-1s could provide more crucial information regarding carcinogenesis. For instance, the percentage of hypomethylation loci (%^u^C^u^C) had a value that could significantly distinguish between normal peripheral blood mononuclear cells (PBMCs) and PBMCs from patients with cancers of the oral cavity, liver, colon, lung and the nasopharynx [41,42]. In this regard, no study has been carried out to analyze LINE-1 and Alu element methylation in human MEC. Thus, the goal of this study was to investigate levels and patterns of LINE-1 and Alu element methylation in MEC and also in the three cell types that are affected by this malignancy. The relationship of methylation status and histological grade in MEC was also assessed to obtain a better understanding of the clinical behavior of the tumor. Here, we demonstrate the methylation level of LINE-1 was different among the three histological grades of mucoepidermoid carcinoma.

## Methods

### Samples and LCM

The research protocol together with the experimental design underwent approval by the Institutional Review Board of the Faculty of Medicine, Chulalongkorn University (IRB006/53). Paraffin-embedded tissues from 24 salivary glands from MEC patients (diagnosed by histology) and 14 normal salivary glands from unrelated patients were obtained from the Department of Pathology, Faculty of Medicine, Chulalongkorn University. The limited clinical data available for each MEC patient was obtained from records, and this is shown in Table 1. The MEC group consisted of 14 women and 10 men (mean age ± SD = 39.62 ± 12.37 years).

**Table 1:** Demographic data of MEC patients

**Sample**	**Sex**	**Age**	**Grade**	**Site**	**Cell type**			
					**N**	**I**	**M**	**S**
**MEC1**	M	60	Low	Palate			√	√
**MEC2**	F	30	Low	Palate	√		√	
**MEC3**	M	35	Low	Palate		√	√	
**MEC4**	M	47	High	Palate	√			√
**MEC5**	F	38	Low	Palate	√			
**MEC6**	M	31	Low	Palate	√	√	√	
**MEC7**	F	32	Low	Palate			√	
**MEC8**	F	53	High	Anterior mandible				√
**MEC9**	M	41	Low	Palate	√		√	
**MEC10**	F	43	Low	Palate			√	√
**MEC11**	M	33	Low	Palate			√	
**MEC12**	F	55	Intermediate	Palate	√			√
**MEC13**	M	54	Low	Palate			√	√
**MEC14**	F	34	Intermediate	Palate				√
**MEC15**	M	35	Low	Palate			√	
**MEC16**	F	16	Intermediate	Palate	√		√	√
**MEC17**	F	21	Intermediate	Palate			√	√
**MEC18**	F	45	Intermediate	Palate		√		√
**MEC19**	F	51	High	Parotid gland	√			√
**MEC20**	M	31	Intermediate	Parotid gland	√		√	
**MEC21**	F	53	Intermediate	Parotid gland	√		√	√
**MEC22**	F	17	Low	Palate	√			
**MEC23**	F	41	Intermediate	Palate	√	√	√	√
**MEC24**	M	55	Intermediate	Palate			√	

MEC: Mucoepidermoid carcinoma, M: Male, F: Female, Low: Low-grade MEC, Intermediate: Intermediate-grade MEC,

High: High-grade MEC, N: Adjacent normal salivary gland cell, I: Intermediate cell, M: Mucous cell, S: Squamous cell.

These specimens were cut into 3-μm-thick sections and mounted onto histological glass slides. After deparaffinization, and hydration, the sections underwent standard hematoxylin and eosin (H&E) staining. After, each slide underwent, histopathological evaluation by three independent pathologists (SK, KD and NK), and those cases correctly identified as MECs were histologically graded according to the WHO diagnostic criteria [1]. The MEC samples assessed yielded low (n=12), intermediate (n=9) and high-grade (n=3) samples based on the 5 histological features (the presence of a cystic component, neural invasion, necrosis, mitotic activity and anaplasia) [1,6,43,44]. For the control group, normal salivary gland tissues were obtained (n=14) from patients undergoing radical neck dissections. All of the normal salivary glands were confirmed by histological analysis to be free of tumor cells.

MEC tissues underwent laser capture microdissection (LCM) using the method described in our previous study [23]. Using our expertise in LCM, we isolated pure cell population of different MEC subtype, as well as normal salivary gland cells adjacent to the lesion. From 24 MEC samples, cell subtypes isolated included squamous (n=13), intermediate (n=4), mucous (n=16), and adjacent normal salivary gland (n=12). Approximately 1,500 cells were isolated from each specimen and used for DNA extraction to yield sufficient amount and quality for PCR analysis (Table 1).

### DNA extraction

DNA was extracted from laser-captured microdissected tissue by proteinase K digestion and a standard phenol-chloroform extraction protocol [45]. For whole MEC tissue anaysis, the paraffin-embedded tissues were cut into 4-μm-thick sections, and DNA was extracted using a DNA extraction kit (QIAamp® DNA FFPE Tissue, Qiagen, Valencia, CA, USA), and the method described previously [46]. The quality of DNA was assessed by NANO Drop 2000C, spectrophotometer with ratio of 1.8-2.0.

### Combine Bisulfite Restriction Analysis (COBRA) of LINE-1 and Alu element

All DNA samples were treated with sodium bisulfite essentially following guidelines provided (EZ DNA Methylation-Gold™ Kit, Zymo research corp, Orange, CA, USA). For COBRALINE-1, the bisulfate-treated DNA was subjected to 40 PCR cycles with LINE-1-F (5’-CCGTAAGGGGTTAGGGAGTTTTT-3’) and LINE-1-R (5’-RTAAAACCCTCCRAACCAAATATAAA-3’) primers at an annealing temperature of 50°C. For COBRAAlu, the bisulfite-treated DNA was subjected to 40 cycles of PCR with two primers, Alu-F (5’-GGCGCGGTGGTTTACGTTTGTAA-3’) and Alu-R (5’-TTAATAAAAACGAAAT TTCACCATATTAACCAAAC-3’) at an annealing temperature of 53°C. After PCR amplification, the LINE-1 amplicons (160 bp) were digested with *TaqI* and *TasI* in NEB buffer 3 (New England Biolabs, Ontario, Canada), while the Alu amplicons (117 bp) were digested with *TaqI* in *TaqI* buffer (MBI Fermentas, Burlington, Canada). Both digestion reactions were incubated at 65°C overnight. The LINE-1 and Alu element digested products were then electrophoresed on an 8% non-denaturing polyacrylamide gel and stained with the SYBR green nucleic acid gel stain (Gelstar, Lonza, Rockland, ME, USA). Distilled water was used as negative control. All experiments were performed in duplicate.

### LINE-1 methylation analysis

**Figure 1:**
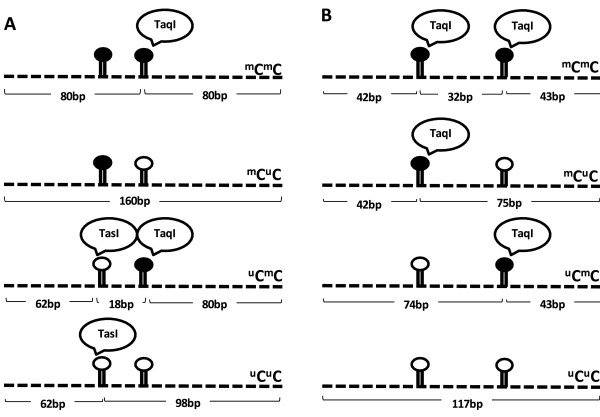
**LINE-1 and Alu methylation patterns.** The dark circles represent methylated cytosine, while the hollow circles represent unmethylated cytosine. There are four possible methylation patterns for the LINE-1 and Alu amplicons, including hypermethylated loci (^m^C^m^C), hypomethylated loci (^u^C^u^C), and 2 partially methylated loci (^m^C^u^C and ^u^C^m^C). In each model, *TaqI* specifically identified methylated cytosine, while *TasI* specifically identified unmethylated cytosine. (**A**) The different methylation patterns of LINE-1 resulted in four differently sized digested products of 160 bp, 98 bp, 80 bp and 62 bp. (**B**) The different methylation patterns of the Alu element resulted in four differently sized digested products of 117 bp, 74/75 bp, 42/43 bp and 32 bp.

The intensities of the COBRALINE-1 fragments on the polyacrylamide gel were quantified and analyzed using a Phosphoimager and the ImageQuant Software (Molecular-Dynamics, GE Healthcare, Slough, UK). COBRALINE-1 generated 4 products depending on the methylation state of the 2 CpG dinucleotides, as follows: partial methylation (^m^C^u^C, 160 bp), hypomethylation (^u^C^u^C, 98 bp), 1 methylated CpG (^m^C, 80 bp) and 1 unmethylated CpG (^u^C, 62 bp) (Figure 1A). LINE-1 methylation levels and patterns were calculated to determine the precise percentage of methylated CpG dinucleotides. The percentage was calculated as follows. First, the intensity of each band was divided by the length (bp) of the double-stranded DNA: %160/160 = A, %98/94 = B, %80/78 = C and %62/62 = D. Next, the frequency of each methylation pattern was calculated: percentage of ^m^C = 100×(C+A)/(C+A+A+B+D), percentage of ^m^C^u^C = 100×(A)/(((C-D+B)/2)+A+D), percentage of ^u^C^m^C = 100×(D-B)/(C-D+B)/2)+A+D, percentage of hypomethylated loci (^u^C^u^C) = 100×B/(((C-D+B)/2)+A+D) and percentage of hypermethylated loci (^m^C^m^C) = 100×((C-D+B)/2)/(((C-D+B)/2)+D+A). DNA samples isolated from HeLa, Jurkat and Daudi cell lines were used as positive controls in each experiment and for interassay variation normalization [21].

### Alu element methylation analysis

The ImageQuant Software (Molecular-Dynamics) was used to quantify the intensities of COBRAAlu fragments on the polyacrylamide gel. COBRAAlu generated 3 bands based on the methylation status: hypomethylation (^u^C^u^C, 117 bp), partial methylation (^m^C^u^C and ^u^C^m^C, 74 and 75 bp, respectively) and methylated loci (^m^C, 42 and 43 bp) (Figure 1B). Alu element methylation levels and patterns were calculated to determine the precise frequency of each pattern. The calculation was performed as the follows. First, the intensity of each band was divided by the length (bp) of the double-stranded DNA: %117/117 = A, %74 and 75/74.5 = B, %42 and 43/43.5 = D, and D-B = C (C= hypermethylated loci, ^m^C^m^C). Next, the frequency of each Alu element methylation pattern was calculated as follows: percentage of methylated loci (^m^C) = 100×(2C+2B)/(2A+2B+2C) = 100×(2D)/(2A+2D), percentage of hypermethylated loci (^m^C^m^C) = 100× C**/**(A+B+C), percentage of partially methylated loci (^u^C^m^C+^m^C^u^C) = 100×B**/**(A+B+C) and percentage of hypomethylated loci (^u^C^u^C) = 100×A**/**(A+B+C). DNA samples from HeLa, Jurkat and Daudi cell lines were used as positive controls in every experiment and to standardize interassay variation [21].

**Figure 2:**
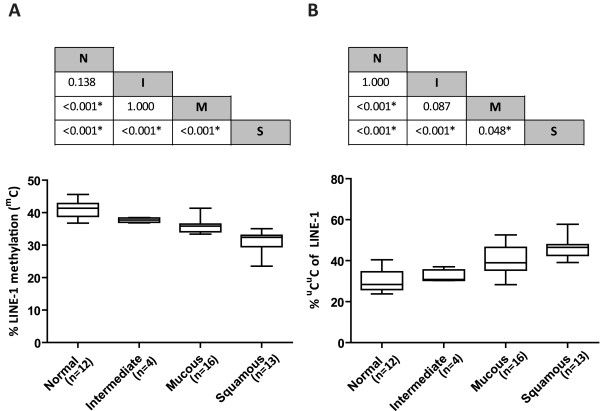
**Comparison of the frequency of total LINE-1 methylation (**^**m**^**C) and **^**u**^**C**^**u**^**C of LINE-1s among MEC cell subtypes.** (**A**) The frequency of ^m^C of LINE-1s among MEC cell subtypes showed a stepwise decrease from normal cells (N) to intermediate cells (I), mucous cells (M) and squamous cells (S). The *p*‐value between each group is shown in the table above the graph. (**B**) The frequency of ^u^C^u^C of LINE-1s among cell types showed a stepwise increase from normal cells to intermediate cells, mucous cells and squamous cells. The *p*‐value between each group is shown in the table above the graph.

### Statistical analysis

Analysis of variance (ANOVA) was used to compare methylation patterns of LINE-1 and Alu elements among squamous, mucous, intermediate and adjacent normal salivary gland cells present in MEC lesions, as well as a paired *t*-test to analyze among cell subtypes in paired samples. An independent sample *t*-test was performed to determine differences between LINE-1 and Alu element methylation patterns in total MEC tissue and normal tissue of the salivary gland. A receiver operating characteristic (ROC) analysis was performed to verify the ability of COBRALINE-1 and COBRAAlu to differentiate MEC lesions from normal salivary gland tissue. An area under the ROC curve (AUC) value of 1.0 indicated perfect accuracy, while an AUC value of 0.5 indicated an inability to distinguish between samples. The cut-off values were selected to determine the diagnostic value of this approach. All calculations were performed using the SPSS software for Windows, version 17.0 (SPSS Inc., Chicago, IL) and the MedCalc statistical software. The results were considered statistically significant when the *p*-value was less than 0.05.

## Results

### LINE-1 methylation in microdissected MEC tissue

The frequency of each LINE-1 methylation pattern is shown in Table 2. The total LINE-1 methylation level (^m^C) decreased from the adjacent normal salivary gland cells (N) to the intermediate cells (I), mucous cells (M) and squamous cells (S). The results showed significant differences between S:M, S:I, S:N and M:N (*p*<0.001). However, there was no significant difference between M:I and N:I (*p*=1.000 and 0.138, respectively) (Figure 2A).

**Table 2:** Frequency of LINE-1 methylation patterns in MEC cell subtypes, whole MEC tissues and normal salivary glands

**LINE-1 patterns**	**%**^**m**^**C**	**%**^**m**^**C**^**m**^**C**	**%**^**m**^**C**^**u**^**C**	**%**^**u**^**C**^**m**^**C**	**%**^**u**^**C**^**u**^**C**
Adjacent normal salivary gland cell (N)	41.13±2.51	12.49±4.61	26.21±4.62	31.06±7.35	30.22±5.08
Intermediate cell (I)	37.69±0.69	7.63±3.15	27.17±0.50	32.94±5.92	32.24±3.20
Mucous cell (M)	35.84±2.24	11.98±7.93	22.90±6.43	24.80±9.75	40.30±6.92
Squamous cell (S)	31.27±3.07	8.74±5.20	24.17±4.00	20.89±8.10	46.18±4.75
Normal salivary gland (NG)	41.79±1.90	21.03±2.31	28.13±2.95	13.38±3.26	37.44±2.86
Whole MEC tissue (MEC)	35.69±2.23	11.52±4.71	26.64±3.20	21.69±6.96	40.13±3.71

Additionally, the frequency of unmethylated (^u^C^u^C) LINE-1s increased from N to M, I and S. Significant differences were found between S:M (*p*=0.048), S:I, S:N and M:N (*p*<0.001). However, no significant difference was found between M:I and N:I (*p*=0.087 and 1.000, respectively) (Figure 2B). A significant difference in the ^u^C^m^C level of LINE-1s in N, M, I and S was found only between S:N (*p*=0.027). There was no significant difference between S:I (*p*=0.099), M:I (*p*=0.551), M:N (*p*=0.353), S:M and N:I (*p*=1.000).

The paired comparisons among cell types displayed strongly significant difference (*p*<0.001) of ^m^C between N:M, N:S and M:S. Furthermore, the ^u^C^u^C is also different when compared between N:M (*p*=0.013), N:S (*p*<0.001) and I:M (*p*=0.006). The detailed data of paired comparisons are shown in Additional file 1: Table S1.

### Alu element methylation in microdissected MEC tissue

**Figure 3:**
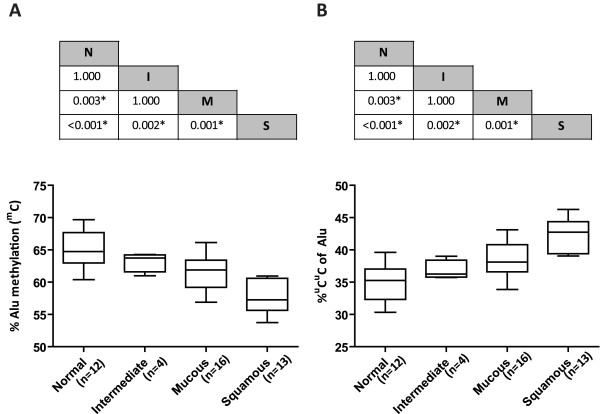
**Comparison of the frequency of total Alu element methylation (**^**m**^**C) and **^**u**^**C**^**u**^**C of Alu elements among MEC cell subtypes.** (**A**) Alu element methylation among cell types showed a stepwise decrease from normal cells to intermediate cells, mucous cells and squamous cell. The *p*‐value between each group is shown in the table above the graph. (**B**) The frequency of ^u^C^u^C of Alu elements among cell types showed a stepwise increase from normal cells to intermediate cells, mucous cells and squamous cells. The *p*‐value between each group is shown in the table above the graph.

The frequency of each Alu element methylation pattern is shown in Table 3. Similar to LINE-1, total Alu element methylation (^m^C) decreased from N to M, I and S. The results showed significant differences between S:M (*p*=0.001), S:I (*p*=0.002), S:N (*p*<0.001) and M:N (*p*=0.003). However, there was no significant difference between M:I and N:I (*p*=1.000) (Figure 3A).

**Table 3:** Frequency of Alu element methylation patterns in MEC cell subtypes, whole MEC tissues and normal salivary glands

**Alu patterns**	**%**^**m**^**C**	**%**^**m**^**C**^**m**^**C**	**%**^**m**^**C**^**u**^**C+**^**u**^**C**^**m**^**C**	**%**^**u**^**C**^**u**^**C**
Adjacent normal salivary gland cell (N)	65.10±2.80	23.36±6.42	41.74±4.43	34.89±2.80
Intermediate cell (I)	63.18±1.51	23.66±10.76	39.52±9.32	36.81±1.51
Mucous cell (M)	61.48±2.46	21.02±6.83	40.45±7.09	38.51±2.46
Squamous cell (S)	57.51±2.46	23.74±5.25	33.77±4.39	42.48±2.46
Normal salivary gland (NG)	64.52±4.66	18.53±10.16	45.99±8.97	35.47±4.66
Whole MEC tissue (MEC)	57.49±5.35	22.21±5.13	35.27±5.02	42.51±5.35

On the contrary, the frequency of ^u^C^u^C of Alu elements increased from N to M, I and S, respectively. A significant difference was found between S:M (*p*=0.001), S:I (*p*=0.002), S:N (*p*<0.001) and M:N (*p*=0.003). No significant difference was found between M:I and N:I (*p*=1.000) (Figure 3B).

A significant difference in the percentage of ^m^C^u^C + ^u^C^m^C of Alu elements was found between S:M (*p*=0.028) and S:N (*p*=0.011). However, there was no significant difference between S:I (*p*=0.061), M:N, M:I and N:I (*p*=1.000). Moreover, the frequency of ^m^C^m^C of Alu elements showed no significant difference between groups of microdissected cells.

For the paired comparisons, significant differences in both ^m^C and ^u^C^u^C of Alu elements were observed as followed: N:M (*p*=0.032), N:S (*p*<0.001) and I:M (*p*=0.008). The detailed data of paired comparisons are shown in Additional file 1: Table S1.

### LINE-1 and Alu element methylation in MECs of various histological grades

**Figure 4:**
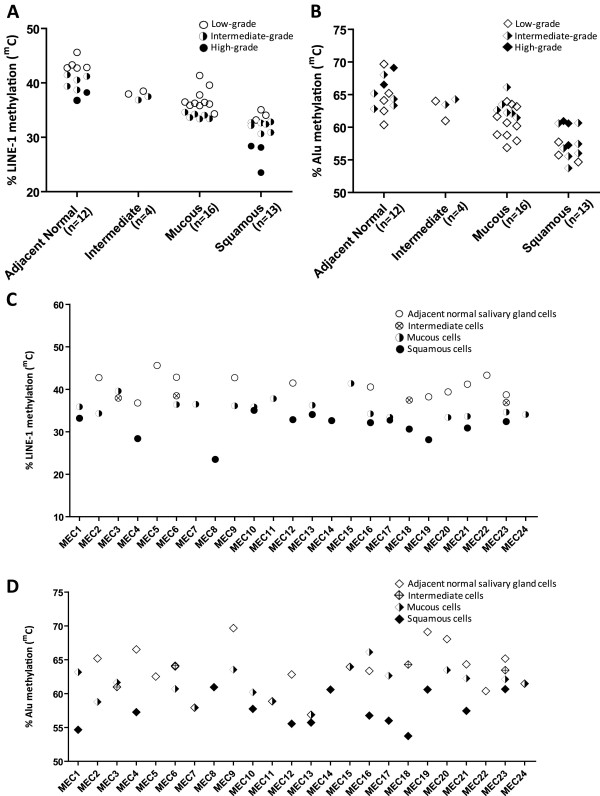
**LINE-1 and Alu element methylation levels among MEC cell subtypes.** (**A**) LINE-1 methylation in MEC cell subtypes correlated with the histological grade of the MEC. (**B**) Alu element methylation level in MEC cell subtypes did not correlate with the histological grade of the MEC. (**C**) LINE-1 methylation level of each microdissected MEC specimen. (**D**) Alu element methylation level of each microdissected MEC specimen.

Total LINE-1 methylation levels (^m^C) of microdissected cells in each cell type decreased from low-grade to intermediate-grade and high-grade MEC, *p*<0.001 (Figure 4A). However, total Alu element methylation (^m^C) in microdissected cells was not related to the histological grade of the MEC (Figure 4B). Interestingly, when we compared the total LINE-1 and Alu element methylation levels of microdissected cells in each specimen, more than 80% of the cases showed decreasing levels of LINE-1 and Alu element methylation from N to I, M and S. (Figure 4C, D). These results demonstrate that genomic hypomethylation, and specifically LINE-1 methylation, essentially correlates with poorer histological grade of MECs.

### LINE-1 and Alu element methylation in whole MEC tissue

We next asked whether these methods could be used to detect and correctly classify MECs. To address this question, we analyzed LINE-1 and Alu element methylation in whole MEC tissues, and compared with normal salivary gland material.

**Figure 5:**
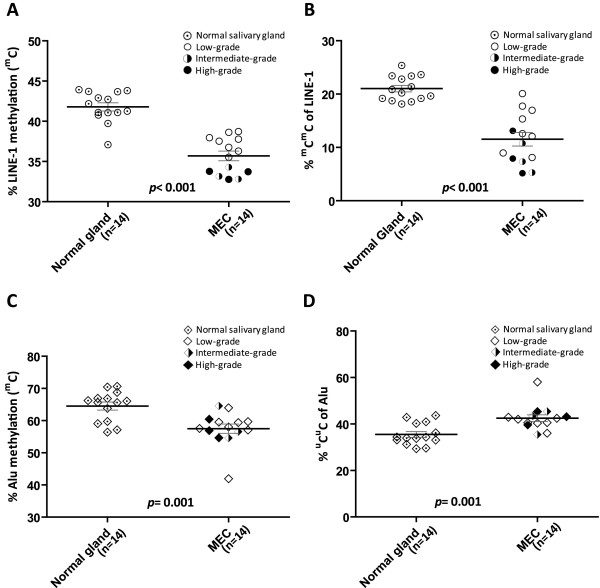
**Comparison of total LINE-1 and Alu element methylation between normal salivary gland tissue and whole MEC tissue.** (**A**, **B**) The frequency of ^m^C and ^m^C^m^C of LINE-1 methylation in whole MEC tissue was significantly lower than in normal salivary gland tissue (p<0.001). (**C**) The frequency of ^m^C of Alu elements in whole MEC tissue was significantly lower than in normal salivary gland tissue (*p*=0.001). (**D**) The frequency of ^u^C^u^C Alu elements in whole MEC tissue was significantly higher than in normal salivary gland tissue (*p*=0.001).

The frequency of each LINE-1 methylation pattern in whole MEC tissue is shown in Table 2. The frequency of ^m^C and ^m^C^m^C LINE-1s were significantly lower in MEC tissue than in normal salivary gland tissue (*p*<0.001) (Table 2, Figure 5A and B). Moreover, the frequency of ^m^C of LINE-1s in the low-grade MECs was higher than in intermediate-grade (*p*<0.001) and high-grade MECs (*p*<0.001), respectively (Figure 5A and B).

The frequency of each Alu element methylation pattern in whole MEC tissue is shown in Table 3. Similar to LINE-1, the total Alu element methylation level (^m^C) in MEC tissue was also significantly lower than in normal salivary gland tissue (*p*=0.001). In agreement with these results, the frequency of ^u^C^u^C of Alu elements in MEC tissue was significantly higher than in normal salivary gland tissue (*p*=0.001) (Table 3, Figure 5C and D). However, Alu element methylation in whole MEC tissue was not related to the histological grade of the MEC (Figure 5C and D).

### Receiver operating characteristic (ROC) analysis of LINE-1 and Alu element methylation

**Figure 6:**
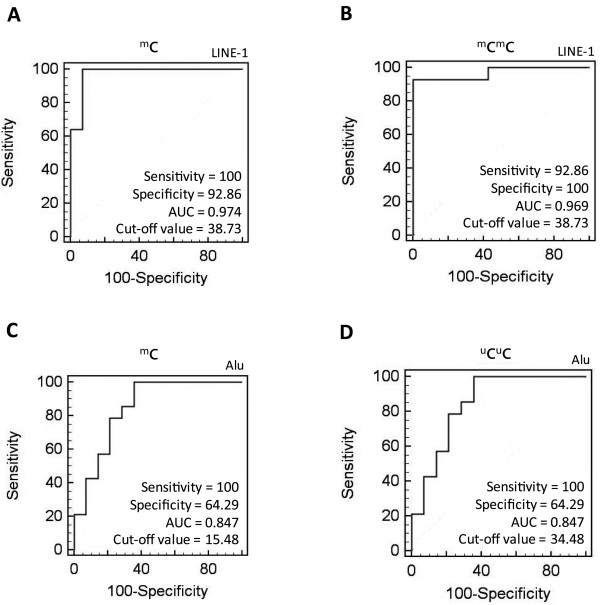
**ROC curve analysis of LINE-1 and Alu element methylation for MEC detection.** (**A**) The total LINE-1 methylation level (^m^C). (**B**) The ^m^C^m^C level of LINE-1 methylation. (**C**) The total Alu element methylation level (^m^C). (**D**) The ^u^C^u^C level of Alu element methylation.

Next, we assessed the ability of these methods to discriminate between MEC tissue and normal salivary gland tissue using an ROC analysis. Among the various patterns of LINE-1 methylation, both the ^m^C and the ^m^C^m^C patterns yielded ROC values indicative of diagnostic reliability. For the ^m^C pattern of LINE-1, the area under the ROC curve (AUC) value was 0.974, while the cut-off value, sensitivity and specificity were 38.73%, 100% and 92.86%, respectively (Figure 6A). The AUC value of the ^m^C^m^C pattern of LINE-1 was 0.969, while the cut-off value, sensitivity and specificity were 38.73%, 92.86% and 100%, respectively (Figure 6B).

Among the various patterns of Alu element methylation, the ^m^C and ^u^C^u^C patterns demonstrated reasonable diagnostic values. Both the ^m^C and the ^u^C^u^C of Alu element methylation patterns had AUC values, sensitivity and specificity of 0.847, 100% and 64.29%, respectively. The cut-off values for the ^m^C and ^u^C^u^C of Alu element methylation patterns were 15.48% and 34.48%, respectively (Figure 6C and D). These results indicate that ROC analysis of LINE-1 methylation may have a stronger diagnostic value than analysis of Alu element methylation. This analysis is especially more effective when both the ^m^C and ^m^C^m^C patterns of LINE-1 methylation are assessed.

## Discussion

To the best of our knowledge, this report represents the first epigenetic study of human MEC. We characterized the methylation status of the repetitive sequences in clinical samples of MEC, and our results clearly show that LINE-1 hypomethylation is in concordant with a poorer histological grade. The COBRA technique represents an excellent approach for detecting the methylation status [41,47], and using for example COBRALINE-1 and COBRAAlu, both are effective in detecting genome-wide methylation status of LINE-1s and Alu elements, respectively, in genomic DNA [48]. In our study, we used a modified method for COBRALINE-1 and COBRAAlu assessing the methylation status, as shown in Figure 1. These methods detected 2 CpG dinucleotide sites and can explain not only methylation level but also methylation patterns which was not reveal by pyrosequencing technique [41].

Although MEC is the most common salivary gland cancer, the overall incidence of occurrence in human is extremely low and thus rarely diagnosed. Therefore, one of the limitations of our study is the small number of MEC samples available for investigation. Theoretically, the parotid gland is the most common site of this tumor; however most of MEC samples used in this study were collected from the minor salivary glands of the palate. In this context, to maximize the number of samples available for analysis, we used laser capture microdissection (LCM), essentially as we have previously shown that this is a very sensitive method for isolating a minimal number cells of interest from whole tissue sections and performing molecular analysis on the extracted nucleic acid. In this study, we procured ~1,500 microdissected cells, which provided sufficient DNA to allow the detection of LINE-1 and Alu methylation levels and pattern. However, LCM did not allow the sufficient isolation of all three cell types from most of the MEC cases, but with some exceptions, for example cases MEC23 (Figure 4C and D), we could efficiently collected every cell population for analysis.

From our data we found that LINE-1 and Alu methylation levels were different among the three histological grades of MEC. We also observed that LINE-1 hypomethylation in adjacent normal salivary gland cells was dependent on the histological grade of the MEC (Figure 4A). This appearance may be explained by some signaling proteins released by cancer cells that can have an influence on normal surrounding tissue in the nearby vicinity [49]. Moreover, the level of LINE-1 methylation in intermediate cells was between that of adjacent normal salivary glands and mucous cells which seem to be correlated with the hypothesis proposed by Luna (2006). According to this author, the intermediate cells, which are derived from reserve cells of salivary duct unit, are believed to be the progenitor cells of the other three cell types of MEC (mucous cells, epidermoid cells and clear cells), and thus they may represent cells in halfway of differentiation between normal reserve cells and the other three cell types of MEC [50]. Since we could not measure the methylation level directly from the reserve cells as they are extremely hard to be identified by microscopic examination, we cannot conclude that the LINE-1 methylation level decrease along the pathway of cell differentiation from the reserve cells of salivary duct unit to the other three cell types of MEC as proposed by Luna (2006).

In conclusion, our findings provide preliminary information of methylation levels between different cell components in MEC, which may be related to histological grading and prognosis of the neoplasm. The knowledge may be applied as a diagnostic tool or a prognostic marker for these tumors in addition to histological grading.

## Abbreviations

MEC: Mucoepidermoid carcinoma; LINE-1: Long INterspersed Element-1; LCM: Laser capture microdissection; COBRA: Combine Bisulfite Restriction Analysis.

## Competing interests

The authors declare that they have no competing interests.

## Authors’ contributions

PS carried out all experiments, performed the statistical analysis and drafted the manuscript. SK and KD participated in the patient enrollment. AM, KS and NK conceived the study, participated in its design and coordination, and revised the manuscript. All authors read and approved the final manuscript.

## Pre-publication history

The pre-publication history for this paper can be accessed here:

http://www.biomedcentral.com/1472-6890/13/10/prepub

## Supplementary Material

Additional file 1: Table 1 Paired comparison of LINE-1 and Alu methylation patterns among MEC cell subtypes. Click here for file
