# Supplementary material for: LINE-1 and Alu hypomethylation in mucoepidermoid carcinoma
Source: BMC Clin Pathol. 2013 Mar 19;13:10. doi: 10.1186/1472-6890-13-10 (PMC3610265; doi:10.1186/1472-6890-13-10)
Supplement: Additional file 1: Table 1 — Paired comparison of LINE-1 and Alu methylation patterns among MEC cell subtypes. [file 1472-6890-13-10-S1.pdf]

Supplement table 1. Paired comparison of LINE-1 and Alu methylation patterns among MEC cell subtypes.

**LINE-1 methylation**		
$\%^{m}C$	N:I	Not analysed
	N:M	$p<0.001$
	N:S	p<0.001
	I:M	$p=0.555$
	I:S	Not analysed
	M:S	$p<0.001$
$\%^{m}C^{m}C$	N:I	Not analysed
	N:M	$p=0.286$
	N:S	$p=0.198$
	I:M	$p=0.155$
	I:S	Not analysed
	M:S	$p=0.935$
$\%^{m}C^{u}C$	N:I	Not analysed
	N:M	$p=0.037$
	N:S	$p=0.097$
	I:M	$p=0.674$
	I:S	Not analysed
	M:S	$p=0.270$
$\%^{u}C^{m}C$	N:I	Not analysed
	N:M	$p=0.542$
	N:S	$p=0.043$
	I:M	$p=0.081$
	I:S	Not analysed
	M:S	p=0.408
$\%^{u}C^{u}C$	N:I	Not analysed
	N:M	$p=0.013$
	N:S	$p<0.001$
	I:M	$p=0.006$
	I:S	Not analysed
	M:S	$p=0.310$

**Alu methylation**		
$\%^{m}C$	N:I	Not analysed
	N:M	$p=0.032$
	N:S	$p<0.001$
	I:M	$p=0.368$
	I:S	Not analysed
	M:S	$p=0.008$
$\%^{m}C^{m}C$	N:I	Not analysed
	N:M	$p=0.313$
	N:S	$p=0.631$
	I:M	$p=0.850$
	I:S	Not analysed
	M:S	$p=0.738$
$\%^{m}C^{u}C+^{u}C^{m}C$	N:I	Not analysed
	N:M	$p=0.937$
	N:S	$p=0.210$
	I:M	$p=0.563$
	I:S	Not analysed
	M:S	$p=0.283$
$\%^{u}C^{u}C$	N:I	Not analysed
	N:M	$p=0.032$
	N:S	$p<0.001$
	I:M	$p=0.368$
	I:S	Not analysed
	M:S	$p=0.008$

MEC: Mucoepidermoid carcinoma
N: Adjacent normal salivary gland cell
I: Intermediate cell
M: Mucous cell
S: Squamous cell
